# Supplementary material for: Heparin-based hydrogel scaffolding alters the transcriptomic profile and increases the chemoresistance of MDA-MB-231 triple-negative breast cancer cells
Source: Biomater Sci. 2020 Feb 13;8(10):2786–96. doi: 10.1039/c9bm01481k (PMC7497406; doi:10.1039/c9bm01481k)
Supplement: Supplementary file 2 [file BM-008-C9BM01481K-s002.zip › Supplementary File 4/HP-B hydrogel + EGF (s)vControl/Pathways/my_analysis.Gsea.1545201170987/HALLMARK_UV_RESPONSE_UP.html]

Details for gene set HALLMARK\_UV\_RESPONSE\_UP[GSEA]

|  || Dataset | expr.class.cls#HYDEGF\_versus\_CONTROL.class.cls#HYDEGF\_versus\_CONTROL\_repos |
| Phenotype | class.cls#HYDEGF\_versus\_CONTROL\_repos |
| Upregulated in class | CONTROL |
| GeneSet | HALLMARK\_UV\_RESPONSE\_UP |
| Enrichment Score (ES) | -0.21768911 |
| Normalized Enrichment Score (NES) | -0.9867431 |
| Nominal p-value | 0.46134022 |
| FDR q-value | 0.5280756 |
| FWER p-Value | 1.0 |
Table: GSEA Results Summary

  

Fig 1: Enrichment plot: HALLMARK\_UV\_RESPONSE\_UP      
 Profile of the Running ES Score & Positions of GeneSet Members on the Rank Ordered List

  

| PROBE | DESCRIPTION (from dataset) | GENE SYMBOL | GENE\_TITLE | RANK IN GENE LIST | RANK METRIC SCORE | RUNNING ES | CORE ENRICHMENT || 1 | FOSB | na |  |  | 0 | 4.410 | 0.0404 | No |
| 2 | RASGRP1 | na |  |  | 87 | 2.987 | 0.0633 | No |
| 3 | CYP1A1 | na |  |  | 102 | 2.902 | 0.0892 | No |
| 4 | TYRO3 | na |  |  | 362 | 2.294 | 0.0966 | No |
| 5 | ATF3 | na |  |  | 473 | 2.154 | 0.1106 | No |
| 6 | CHKA | na |  |  | 840 | 1.788 | 0.1078 | No |
| 7 | NXF1 | na |  |  | 950 | 1.717 | 0.1178 | No |
| 8 | CLTB | na |  |  | 1205 | 1.566 | 0.1189 | No |
| 9 | ACAA1 | na |  |  | 1222 | 1.561 | 0.1324 | No |
| 10 | ASNS | na |  |  | 1455 | 1.448 | 0.1335 | No |
| 11 | SULT1A1 | na |  |  | 1665 | 1.367 | 0.1350 | No |
| 12 | NR4A1 | na |  |  | 1977 | 1.273 | 0.1304 | No |
| 13 | CLCN2 | na |  |  | 2162 | 1.222 | 0.1320 | No |
| 14 | TCHH | na |  |  | 2179 | 1.217 | 0.1423 | No |
| 15 | FOS | na |  |  | 2215 | 1.207 | 0.1515 | No |
| 16 | TGFBRAP1 | na |  |  | 2401 | 1.150 | 0.1524 | No |
| 17 | MARK2 | na |  |  | 2412 | 1.148 | 0.1624 | No |
| 18 | CEBPG | na |  |  | 2757 | 1.062 | 0.1541 | No |
| 19 | CCNE1 | na |  |  | 3012 | 1.001 | 0.1499 | No |
| 20 | DGAT1 | na |  |  | 3215 | 0.956 | 0.1481 | No |
| 21 | POLG2 | na |  |  | 3273 | 0.948 | 0.1538 | No |
| 22 | H2AFX | na |  |  | 3433 | 0.916 | 0.1539 | No |
| 23 | WIZ | na |  |  | 3544 | 0.896 | 0.1563 | No |
| 24 | CDK2 | na |  |  | 3627 | 0.880 | 0.1601 | No |
| 25 | STK25 | na |  |  | 3666 | 0.872 | 0.1661 | No |
| 26 | CDC34 | na |  |  | 3718 | 0.861 | 0.1713 | No |
| 27 | HTR7 | na |  |  | 3954 | 0.820 | 0.1665 | No |
| 28 | STARD3 | na |  |  | 4399 | 0.749 | 0.1501 | No |
| 29 | ARRB2 | na |  |  | 4788 | 0.689 | 0.1360 | No |
| 30 | PRPF3 | na |  |  | 4946 | 0.660 | 0.1339 | No |
| 31 | SHOX2 | na |  |  | 5053 | 0.645 | 0.1342 | No |
| 32 | C4BPB | na |  |  | 5314 | 0.603 | 0.1261 | No |
| 33 | GLS | na |  |  | 5355 | 0.596 | 0.1295 | No |
| 34 | EIF5 | na |  |  | 5751 | 0.537 | 0.1137 | No |
| 35 | PLCL1 | na |  |  | 5947 | 0.510 | 0.1081 | No |
| 36 | CHRNA5 | na |  |  | 6428 | 0.445 | 0.0870 | No |
| 37 | PPIF | na |  |  | 6528 | 0.434 | 0.0858 | No |
| 38 | DDX21 | na |  |  | 6980 | 0.373 | 0.0656 | No |
| 39 | HSPA2 | na |  |  | 7157 | 0.351 | 0.0596 | No |
| 40 | TUBA4A | na |  |  | 7430 | 0.313 | 0.0482 | No |
| 41 | MGAT1 | na |  |  | 7453 | 0.311 | 0.0499 | No |
| 42 | FURIN | na |  |  | 7555 | 0.298 | 0.0473 | No |
| 43 | DLG4 | na |  |  | 7838 | 0.257 | 0.0349 | No |
| 44 | TFRC | na |  |  | 7904 | 0.255 | 0.0338 | No |
| 45 | YKT6 | na |  |  | 8014 | 0.241 | 0.0303 | No |
| 46 | PPAT | na |  |  | 8194 | 0.225 | 0.0230 | No |
| 47 | FEN1 | na |  |  | 8723 | 0.161 | -0.0032 | No |
| 48 | TARS | na |  |  | 8852 | 0.141 | -0.0086 | No |
| 49 | CCND3 | na |  |  | 8864 | 0.140 | -0.0079 | No |
| 50 | AMD1 | na |  |  | 9158 | 0.108 | -0.0223 | No |
| 51 | SIGMAR1 | na |  |  | 9593 | 0.062 | -0.0445 | No |
| 52 | NPTXR | na |  |  | 9695 | 0.050 | -0.0493 | No |
| 53 | CNP | na |  |  | 9702 | 0.050 | -0.0492 | No |
| 54 | RXRB | na |  |  | 9730 | 0.046 | -0.0502 | No |
| 55 | PRKCD | na |  |  | 10287 | -0.019 | -0.0792 | No |
| 56 | HMOX1 | na |  |  | 10412 | -0.037 | -0.0854 | No |
| 57 | ONECUT1 | na |  |  | 10538 | -0.041 | -0.0915 | No |
| 58 | NAT1 | na |  |  | 10635 | -0.057 | -0.0960 | No |
| 59 | PDAP1 | na |  |  | 10816 | -0.080 | -0.1048 | No |
| 60 | AP2S1 | na |  |  | 10871 | -0.089 | -0.1068 | No |
| 61 | IRF1 | na |  |  | 11060 | -0.115 | -0.1156 | No |
| 62 | PARP2 | na |  |  | 11606 | -0.176 | -0.1425 | No |
| 63 | FKBP4 | na |  |  | 11609 | -0.176 | -0.1410 | No |
| 64 | RHOB | na |  |  | 11865 | -0.214 | -0.1524 | No |
| 65 | CYB5R1 | na |  |  | 12283 | -0.259 | -0.1719 | No |
| 66 | POLE3 | na |  |  | 12483 | -0.284 | -0.1798 | No |
| 67 | GRPEL1 | na |  |  | 12492 | -0.285 | -0.1776 | No |
| 68 | PSMC3 | na |  |  | 12722 | -0.318 | -0.1867 | No |
| 69 | POLR2H | na |  |  | 12745 | -0.321 | -0.1849 | No |
| 70 | BAK1 | na |  |  | 12757 | -0.324 | -0.1825 | No |
| 71 | DNAJB1 | na |  |  | 13062 | -0.352 | -0.1952 | No |
| 72 | PPT1 | na |  |  | 13258 | -0.379 | -0.2020 | No |
| 73 | PDLIM3 | na |  |  | 13293 | -0.385 | -0.2002 | No |
| 74 | CDC5L | na |  |  | 13501 | -0.413 | -0.2073 | No |
| 75 | CDKN1C | na |  |  | 13565 | -0.423 | -0.2067 | No |
| 76 | RFC4 | na |  |  | 13625 | -0.429 | -0.2059 | No |
| 77 | APOM | na |  |  | 13636 | -0.431 | -0.2024 | No |
| 78 | RRAD | na |  |  | 13718 | -0.444 | -0.2026 | No |
| 79 | HLA-F | na |  |  | 13768 | -0.453 | -0.2010 | No |
| 80 | PPP1R2 | na |  |  | 13923 | -0.475 | -0.2048 | No |
| 81 | SQSTM1 | na |  |  | 13980 | -0.486 | -0.2032 | No |
| 82 | BTG2 | na |  |  | 14115 | -0.493 | -0.2057 | No |
| 83 | SLC6A8 | na |  |  | 14163 | -0.502 | -0.2036 | No |
| 84 | TST | na |  |  | 14375 | -0.533 | -0.2098 | No |
| 85 | SPR | na |  |  | 14382 | -0.534 | -0.2052 | No |
| 86 | HNRNPU | na |  |  | 14621 | -0.576 | -0.2124 | Yes |
| 87 | TMBIM6 | na |  |  | 14679 | -0.586 | -0.2100 | Yes |
| 88 | BTG3 | na |  |  | 14693 | -0.589 | -0.2053 | Yes |
| 89 | ENO2 | na |  |  | 14703 | -0.591 | -0.2004 | Yes |
| 90 | PRKACA | na |  |  | 14733 | -0.596 | -0.1964 | Yes |
| 91 | GCH1 | na |  |  | 14797 | -0.603 | -0.1942 | Yes |
| 92 | EIF2S3 | na |  |  | 14980 | -0.629 | -0.1980 | Yes |
| 93 | MRPL23 | na |  |  | 15059 | -0.644 | -0.1962 | Yes |
| 94 | AQP3 | na |  |  | 15228 | -0.674 | -0.1988 | Yes |
| 95 | EPHX1 | na |  |  | 15301 | -0.687 | -0.1963 | Yes |
| 96 | RPN1 | na |  |  | 15354 | -0.697 | -0.1926 | Yes |
| 97 | KLHDC3 | na |  |  | 15531 | -0.728 | -0.1952 | Yes |
| 98 | CXCL2 | na |  |  | 15653 | -0.754 | -0.1946 | Yes |
| 99 | ATP6V1C1 | na |  |  | 15672 | -0.757 | -0.1886 | Yes |
| 100 | GRINA | na |  |  | 15854 | -0.792 | -0.1908 | Yes |
| 101 | BSG | na |  |  | 15875 | -0.796 | -0.1846 | Yes |
| 102 | JUNB | na |  |  | 15969 | -0.814 | -0.1820 | Yes |
| 103 | HYAL2 | na |  |  | 16054 | -0.834 | -0.1787 | Yes |
| 104 | SLC25A4 | na |  |  | 16080 | -0.840 | -0.1724 | Yes |
| 105 | ALAS1 | na |  |  | 16248 | -0.886 | -0.1730 | Yes |
| 106 | EPCAM | na |  |  | 16264 | -0.890 | -0.1656 | Yes |
| 107 | NFKBIA | na |  |  | 16276 | -0.892 | -0.1580 | Yes |
| 108 | BCL2L11 | na |  |  | 16361 | -0.913 | -0.1541 | Yes |
| 109 | E2F5 | na |  |  | 16388 | -0.920 | -0.1470 | Yes |
| 110 | STIP1 | na |  |  | 16399 | -0.923 | -0.1390 | Yes |
| 111 | LYN | na |  |  | 16413 | -0.926 | -0.1312 | Yes |
| 112 | ALDOA | na |  |  | 16587 | -0.967 | -0.1314 | Yes |
| 113 | GPX3 | na |  |  | 16764 | -1.015 | -0.1314 | Yes |
| 114 | CYB5B | na |  |  | 16935 | -1.066 | -0.1305 | Yes |
| 115 | TAP1 | na |  |  | 16995 | -1.089 | -0.1236 | Yes |
| 116 | ICAM1 | na |  |  | 17147 | -1.127 | -0.1212 | Yes |
| 117 | BID | na |  |  | 17292 | -1.178 | -0.1180 | Yes |
| 118 | CASP3 | na |  |  | 17375 | -1.207 | -0.1112 | Yes |
| 119 | MAOA | na |  |  | 17610 | -1.299 | -0.1116 | Yes |
| 120 | UROD | na |  |  | 17690 | -1.327 | -0.1035 | Yes |
| 121 | BTG1 | na |  |  | 17748 | -1.358 | -0.0941 | Yes |
| 122 | ATP6V1F | na |  |  | 17866 | -1.424 | -0.0872 | Yes |
| 123 | CREG1 | na |  |  | 18165 | -1.591 | -0.0882 | Yes |
| 124 | IL6 | na |  |  | 18227 | -1.619 | -0.0766 | Yes |
| 125 | RAB27A | na |  |  | 18367 | -1.728 | -0.0680 | Yes |
| 126 | HSPA13 | na |  |  | 18383 | -1.739 | -0.0528 | Yes |
| 127 | IL6ST | na |  |  | 18442 | -1.792 | -0.0395 | Yes |
| 128 | BMP2 | na |  |  | 18629 | -1.962 | -0.0312 | Yes |
| 129 | DNAJA1 | na |  |  | 18633 | -1.967 | -0.0133 | Yes |
| 130 | GGH | na |  |  | 18745 | -2.106 | 0.0001 | Yes |
| 131 | SOD2 | na |  |  | 18995 | -2.569 | 0.0106 | Yes |
Table: GSEA details [plain text format]

  

Fig 2: HALLMARK\_UV\_RESPONSE\_UP      
 Blue-Pink O' Gram in the Space of the Analyzed GeneSet

  

Fig 3: HALLMARK\_UV\_RESPONSE\_UP: Random ES distribution      
 Gene set null distribution of ES for **HALLMARK\_UV\_RESPONSE\_UP**
